# Mechanism of Action Potential Prolongation During Metabolic Inhibition in the Whole Rabbit Heart

**DOI:** 10.3389/fphys.2018.01077

**Published:** 2018-08-09

**Authors:** Regina Mačianskienė, Irma Martišienė, Antanas Navalinskas, Rimantas Treinys, Inga Andriulė, Jonas Jurevičius

**Affiliations:** Institute of Cardiology, Lithuanian University of Health Sciences, Kaunas, Lithuania

**Keywords:** action potential prolongation, transmural APD dispersion, metabolic inhibition, FCCP, whole rabbit heart

## Abstract

Myocardial ischemia is associated with significant changes in action potential (AP) duration, which has a biphasic response to metabolic inhibition. Here, we investigated the mechanism of initial AP prolongation in whole Langendorff-perfused rabbit heart. We used glass microelectrodes to record APs transmurally. Simultaneously, optical AP, calcium transient (CaT), intracellular pH, and magnesium concentration changes were recorded using fluorescent dyes. The fluorescence signals were recorded using an EMCCD camera equipped with emission filters; excitation was induced by LEDs. We demonstrated that metabolic inhibition by carbonyl cyanide-*p*-trifluoromethoxyphenylhydrazone (FCCP) resulted in AP shortening preceded by an initial prolongation and that there were no important differences in the response throughout the wall of the heart and in the apical/basal direction. AP prolongation was reduced by blocking the *I*_CaL_ and transient outward potassium current (*I*_to_) with diltiazem (DTZ) and 4-aminopyridine (4-AP), respectively. FCCP, an uncoupler of oxidative phosphorylation, induced reductions in CaTs and intracellular pH and increased the intracellular Mg^2+^ concentration. In addition, resting potential depolarization was observed, clearly indicating a decrease in the inward rectifier K^+^ current (*I*_K1_) that can retard AP repolarization. Thus, we suggest that the main currents responsible for AP prolongation during metabolic inhibition are the *I*_CaL_, *I*_to_, and *I*_K1_, the activities of which are modulated mainly by changes in intracellular ATP, calcium, magnesium, and pH.

## Introduction

Myocardial ischemia is associated with significant changes in AP duration. Studies on cardiac preparations have shown that myocardial ischemia or treatment with potent mitochondrial inhibitors induces a biphasic response in APD, i.e., the initial prolongation is accompanied by a subsequent shortening of the AP (Verkerk et al., 1996). Since the prolongation and shortening of AP could be distributed spatially and transmurally throughout the heart, it may lead to APD and refractory period dispersion, which could be one of the reasons for the development of dangerous arrhythmias. Therefore, investigations of the mechanisms of the very first minutes of ischemic stress could be of pathophysiological importance.

During metabolic inhibition, almost all inward and outward currents and/or ion exchange systems can be changed (Carmeliet, 1999) due to decreases in intracellular ATP levels, acidification, and increases in intracellular calcium and magnesium concentrations, as well as of other ions, and these changes could induce prolongation and/or shortening of APs. The most important factor responsible for the shortening of APs is the potassium current (*I*_KATP_) which is activated by a reduced intracellular ATP concentration. The prolongation of APs can be produced by increased activity of the inward sodium and calcium currents, by reduced activity of the outward potassium current, or a combination of changes in multiple currents and/or exchangers. It is well-known that during ischemic injury, late sodium current (*I*_NaLate_) activity can be enhanced (Undrovinas and Maltsev, 2008), and the *I*_CaL_ activity can be prolonged due to acidosis (Saegusa et al., 2011) or impaired sarcoplasmic reticulum (SR) function (Sham et al., 1995). Increases in both of these currents may induce AP prolongation.

Many outward ion currents participate in AP formation, but only some of them can be reduced by metabolic inhibition and lead to AP prolongation. It has been reported that during metabolic inhibition the transient outward current (*I*_to_), which is responsible for the early phase of repolarization, is reduced (Ogbaghebriel and Shrier, 1994; Barth and Tomaselli, 2009). The inward rectifier K^+^ current (*I*_K1_)_,_ which is responsible for maintaining the resting potential and participates in AP repolarization, can be reduced as well, mainly due to an increased intracellular free Mg^2+^ concentration (Matsuda et al., 1987). Meanwhile, since there are no data indicating that metabolic inhibition can reduce the main repolarizing delayed rectifying potassium currents (*I*_Kr_ and *I*_Ks_), it is unlikely that they might have any impact on the prolongation of AP.

The detailed ionic mechanisms of the initial prolongation of APs in single rabbit cardiomyocytes during metabolic injury have been investigated previously, and this effect was linked to *I*_to_ changes (Verkerk et al., 1996). This effect was found in all subepicardial (subEpi) cells, where the *I*_to_ has a big impact on APD; however, this phenomenon was almost absent in subendocardial (subEndo) myocytes. Considering that the distinctions between the properties of different types of cardiac cells can be less apparent in the whole heart (Anyukhovsky et al., 1996), we sought to investigate this phenomenon in the entire heart in detail.

In the present study, we investigated the effect of FCCP, an uncoupler of oxidative phosphorylation, on APD in whole Langendorff-perfused rabbit hearts. Our results show that there are no important differences in the biphasic response throughout the wall of the heart and in the apical/basal direction. The data suggest that the main currents responsible for AP prolongation during metabolic inhibition are *I*_CaL_, *I*_to_, and *I*_K1_, the activities of which are modulated mainly by changes in intracellular ATP, calcium, magnesium, and pH.

## Materials and Methods

### Experimental Preparation

All experiments were performed according to the European Community guiding principles and were approved by the State Food and Veterinary Service of the Republic of Lithuania (2015-09-24 No. G2-34).

New Zealand white rabbits (*n* = 20) of either sex (∼3 kg) were used in this study, and the methods were similar to those described in detail previously (Mačianskienė et al., 2015, 2017). Briefly, rabbits were first sedated by subcutaneous injections of xylazine (10 mg/kg), including heparin (1000 U/kg), and were then anesthetized with intravenous injections of ketamine (10 mg/kg). The excised heart was immediately mounted on a Langendorff perfusion setup (ADInstruments, Oxford, United Kingdom) and retrogradely perfused *via* the aorta at a constant pressure (80 mmHg) at 37 ± 0.2°C for 30 min with an oxygenated Tyrode solution (in mmol/L: 135 NaCl, 5.4 KCl, 1.8 CaCl_2_, 0.9 MgCl_2_, 0.33 NaH_2_PO_4_, 10 glucose, and 10 HEPES; pH 7.4, adjusted with NaOH). Then, the perfusion was switched to a recirculation mode and blebbistatin (20 μmol/L) was added to eliminate contractions. After stabilization, the heart was stained by adding of 3 μmol/L voltage-sensitive dye (di-4-ANBDQBS) into the perfusate for recording of optical APs (OAPs) or by a 20-mL bolus injection of SNARF-1 AM ester (SNARF-1, 10 μmol/L) or Rhod-2 AM ester (Rhod-2, 10 μmol/L) for intracellular pH (pH_i_) or Ca^2+^ transient (CaT) measurements, respectively. A 10-mL bolus injection of Magnesium Green AM ester (MgG) (5 μmol/L) or Mitotracker Deep Red (MTDR) (10 μmol/L) was used for intracellular free Mg^2+^ ([Mg^2+^]_i_) or mitochondrial membrane potential measurements, respectively. Metabolic inhibition was induced by FCCP (1 μmol/L).

Endocardial pacing at a 300-ms period was continuously maintained throughout the experiment *via* a bipolar silver electrode inserted into the LV cavity close to apex, with a 2-ms impulse at set twice the threshold.

### Microelectrode Recordings

Action potentials were recorded using glass microelectrodes (filled with 3 mol/L KCl) with long tips (resistance ∼30–50 MΩ), which causes less damage to the tissue during deep intramyocardial recordings. The microelectrodes were inserted in the LV wall (the thickness was ∼5 mm) from the epicardial surface and moved transmurally until they reached the subendocardium (Mačianskienė et al., 2015). The depth of insertion was controlled by hydraulic micromanipulators (Narishige, Japan) and special depth detectors (Millitron, Measuring Probes 1310) with an accuracy of ≤1 μm. The signal from these detectors was recorded simultaneously with the APs. The 0 point for depth measurements was set when the microelectrodes reached the epicardial surface. Recorded APs were amplified and digitized by the 16-channel PowerLab system (ADInstruments, Oxford, United Kingdom) at a frequency of 20 kHz. The data were recorded and analyzed using LabChart 8 Pro software.

### Optical Recordings

The optical mapping setup has been described previously (Mačianskienė et al., 2017). Briefly, for measuring of optical signals from fluorescent dyes, we used a collimated LEDs (all from Thorlabs, United States). Emission was measured using corresponding filters for specific wavelengths with a cooled (-100°C), fast 14-bit EMCCD camera (iXon^EM+^DU-860, Andor Technology, Ireland) equipped with a 50-mm focal length objective (Navitar, United States). Specifically, the dyes were excited with the following LEDs: di-4-ANBDQBS using a 660 nm LED (M660L3, filtered at 650/40), Rhod-2 using a 565 nm LED (M565L3, filtered at 554/23), SNARF-1 using a 505 nm LED (M505L3, filtered at 504/12), MgG using a 505 nm LED filtered at 504/12, and MTDR using a 660 nm LED filtered at 640/25. The emitted fluorescence was filtered with a 720 nm long-pass filter for di-4-ANBDQBS, a 605/55 nm bandpass filter for Rhod-2, both a 590/20 nm and a 640/30 nm bandpass filters for SNARF-1, a 540/25 nm bandpass filter for MgG, and a 680/30 nm bandpass filter for MTDR, which were placed in front of the camera. For OAP and CaT recordings, optical signals of 5.2 s were acquired, and the final 16 signals were averaged. SNARF-1 signals at two fluorescence wavelengths were recorded over 3 s, and during this time, the two emission filters were alternated (switching time was less than 200 ms). Fluorescence from MgG and MTDR was recorded over 3 s. To mark the time of stimulation in the OAP and CaT recordings, an array of small LEDs (500–940 nm) that generated 2-ms pulses in synchrony with the pacing cycle was placed in the camera’s field of view. The anterior surface of the LV was imaged, and the field of view was 20 mm × 20 mm. Videos were acquired at a sampling rate of 500 Hz with a resolution of 128 × 128 pixels using imaging software (Andor SOLIS x-3467). The videos were processed using ImageJ software. All of the experiments were performed under dark conditions.

### Chemicals

The following fluorescent dyes and chemicals were used: di-4-ANBDQBS (JPW-6033) (from Dr. L. Loew, University of Connecticut, United States), Rhod-2 (Biotium, Fremont, CA, United States), SNARF-1 and MTDR (Molecular Probes, United States), MgG (Invitrogen, United States), and (±)-blebbistatin (Cayman, United States). The other chemicals, including FCCP, glibenclamide, 4-AP, DTZ, and oligomycin were purchased from Sigma-Aldrich (United States). Water-insoluble compounds were initially dissolved in dimethyl sulfoxide (DMSO) or ethanol to make stock solutions, which were then diluted. The final concentration of the solvent was <0.1%, and the solvent did not affect the measurements.

### Data Analysis and Statistics

For simultaneously obtained OAPs and microelectrode APs, the activation time was evaluated at 50% of upstroke, and the duration (OAPD and APD) was determined at 20, 50, and 90% of repolarization. OAPD maps were constructed from videos using custom Scroll 1.16 software developed by Dr. S. Mironov (University of Michigan, United States). The maps of OAPD at 50% repolarization (OAPD50) and of OAPD90, showing increases/decreases (i.e., prolongation/shortening), were constructed from calculated and normalized values at every pixel of the mapping area:

$$\begin{array}{c} {\mathrm{OAPD}}_{increase/decrease}\;=\;({\mathrm{OAPD}}_{\mathrm{FCCP}}{{}_{↑}}_{/}{{}_{\mathrm{FCCP}}}_{5^{\prime -}}\;{\mathrm{OAPD}}_{\mathrm{control}})\;\times \;{100/OAPD}_{\mathrm{control}}. \end{array}$$

where FCCP↑ is the time of the maximal prolongation of APD upon FCCP action.

In the same manner, CaT amplitude maps were constructed. The background fluorescence value was subtracted from all optical signals.

Data are presented as the means ± standard errors of the mean (SEM). The significance of differences was evaluated using one-way analysis of variance (ANOVA). In the cases when the effect of FCCP on AP parameters depending on transmural distribution (subEpi/subEndo) or drug presence (DTZ, ranolazine, 4-AP, and oligomycin) was evaluated, the significance of changes was compared between values obtained at the same time of FCCP action. The significance level was set at *p* < 0.05.

## Results

### Effect of FCCP on Action Potential Duration

Ischemia induces APD shortening, which is preceded by an initial prolongation. Earlier investigations of ionic mechanisms related to the initial increase in APD under metabolic inhibition were conducted on single cardiomyocytes (Isenberg et al., 1983; Verkerk et al., 1996), and these studies were probably unable to reveal the associated compensation mechanisms occurring in the whole heart. Therefore, it remained unclear whether the biphasic effect of metabolic inhibition persists in the whole heart. This prompted us to investigate the ionic mechanisms underlying the effects of FCCP, a mitochondrial uncoupler, in Langendorff-perfused rabbit hearts in detail.

The main aim of our study was to investigate the mechanisms of the initial prolongation of AP during metabolic inhibition of the whole rabbit heart. The prolongation of the AP appears at the first minute upon perfusion of the heart with the mitochondrial uncoupler FCCP. At the same time, the shortening of the AP starts because of the activation of ATP-sensitive potassium current (*I*_KATP_). Our preliminary data showed that the prolongation and shortening of AP were of quite different dynamics in different experiments. Therefore, all data in the study were obtained under *I*_KATP_ partial reduction by glibenclamide (10 μmol/L), which helped to observe the stable prolongation of the AP upon metabolic inhibition in all our experiments. Glibenclamide at this concentration had no marked impact on the AP parameters in the control (results not shown) but had the benefit of slowing the shortening of APs and protecting the heart from the early development of ventricular fibrillation in combination with FCCP administration (Smith et al., 2012). Glibenclamide was maintained constantly throughout control treatments and subsequent perfusion with FCCP (1 μmol/L) and other drugs.

**FIGURE 1:**
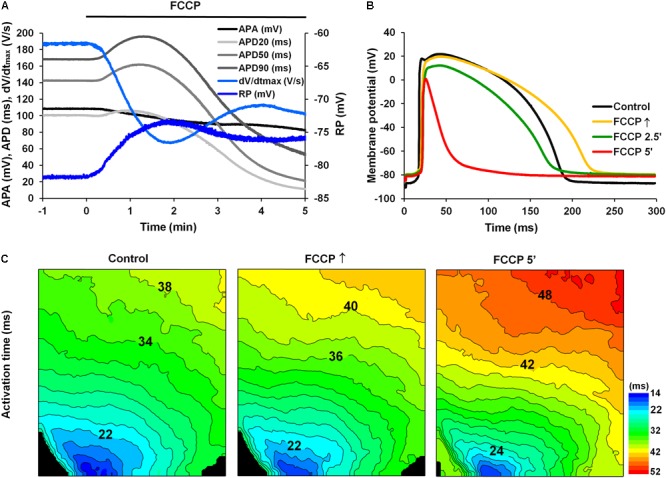
Effect of FCCP (1 μmol/L) on the AP parameters of the left ventricle of Langendorff-perfused rabbit hearts. **(A)** A typical example of time dependent changes in AP parameters (at 2.3 mm depth from the epicardium): the amplitude (APA; *black*); the duration at 20% (APD20; *light gray*), 50% (APD50; *gray*), and 90% (APD90; *dark gray*) of repolarization; the maximal value of the first time derivative of the AP upstroke (dV/dt_max_; *light blue*); and the resting membrane potential (RP, *blue*). FCCP perfusion starts at time 0. Note the biphasic profile of APD changes. **(B)** Superimposition of APs recorded under control conditions (i.e., Tyrode solution containing 10 μmol/L glibenclamide and 20 μmol/L blebbistatin; *black*) and at selected times with the effects of FCCP: at the maximal prolongation of APD (FCCP↑; *yellow*), at 2.5 min (FCCP 2.5′; *green*), and at 5 min (FCCP 5′; *red*) of FCCP treatment. FCCP↑, the time of the maximal prolongation of AP upon FCCP action. Note the lengthening vs. shortening effect at FCCP↑ and FCCP 5′, respectively. **(C)** Optical AP (OAP) activation time maps. Numbers near isolines show the activation time in ms. The interval between isolines on the maps is 2 ms. Note that the direction of movement of the activation wave is from blue-green to red. Stimulation electrodes were located inside the LV (near the apex).

**Figure 1A** shows a typical example of changes in various AP parameters as a function of time during perfusion with FCCP. **Figure 1B** shows overlapped AP traces obtained in responses to the initial and subsequent effects of FCCP on the LV of rabbit hearts at a 300-ms pacing period. It is seen that the effect of FCCP has a biphasic profile, which, after an initial transient prolongation, resulted in marked APD shortening at 20, 50, and 90% of the repolarization (see **Figure 1A**, *light gray, gray*, and *dark gray*, respectively). The time when the maximal prolongation of the AP was registered varied in different experiments; that value is indicated as the time of the maximal prolongation of AP upon FCCP action (FCCP↑), which, on average for subEpi and subEndo cells, equaled 1.11 ± 0.10 and 1.09 ± 0.07 min, respectively (n = 9, for each). In contrast to APD, the other AP parameters, i.e., the APA, the dV/dt_max_, and the RP, gradually decreased due to the effects of FCCP. The partial recovery of dV/dt_max_ during the continuous perfusion of FCCP was possibly conditioned by changes in depolarization of RP and in the biphasic APD since the magnitude of dV/dt_max_ directly depends on the *I*_Na_ and its recovery from inactivation during the diastolic interval. The mean values of AP parameters under the control condition and under the effects of FCCP are presented in **Table 1**. The representative activation time maps of OAPs obtained using the voltage-sensitive dye di-4-ANBDQBS are presented in **Figure 1C**. The maps show that conduction was already slightly slowed down at the beginning of the effects of FCCP, when maximal prolongation of AP was registered (*middle*); at 5 min (*right*) this effect was even more pronounced.

**Table 1:** AP parameters under FCCP (1 μmol/L) treatment (*n* = 18 for each).

	dV/dt_max_ (V/s)	RP (mV)	APA (mV)	APD20 (ms)	APD50 (ms)	APD90 (ms)
Control	126:6 ± 10:0	–77.6 ± 0.5	107.2 ± 1.3	86.4 ± 2.0	130.7 ± 1.7	159.3 ± 1.6
FCCP↑	88.4 ± 7.7*	–72.6 ± 1.0*	94.0 ± 3.3*	90.4 ± 2.0*	142.3 ± 2.5*	178.0 ± 2.7*
FCCP 2.5′	65.4 ± 6.9*	–69.2 ± 1.2*	88.7 ± 3.3*	60.8 ± 3.3*	95.2 ± 5.3*	125.1 ± 6.2*
FCCP 5′	70.6 ± 9.7*	–69.0 ± 1.4*	75.0 ± 6.1*	29.6 ± 4.3*	46.8 ± 6.3*	73.7 ± 6.6*

*Control – Tyrode solution containing glibenclamide (10 μmol/L) and blebbistatin (20 μmol/L), FCCP↑, FCCP 2.5′, and FCCP 5′, FCCP effects at the maximal prolongation of APD, at 2.5 min, and at 5 min, respectively; dV/dt_*max*_, the maximal value of the first time derivative of the AP upstroke; RP, resting membrane potential; APA, AP amplitude; APD20, APD50, and APD90, APD at 20, 50, and 90% of repolarization, respectively. FCCP↑, the time of the maximal prolongation of AP upon FCCP action. Note the averaged depth of microelectrode insertion was 1.78 ± 0.15 mm from the epicardium. Statistical analysis was performed using ANOVA. ^∗^*p* < 0.05 vs. control.*

For confirmation that the biphasic effect elicited by FCCP is related to metabolic inhibition, we tested for changes in mitochondrial membrane potential using the fluorescent MTDR dye. The time-dependent fluorescence changes showing alterations in mitochondrial membrane potential are presented in **Supplementary Figure S1**. A clear and instant decrease in fluorescence was obtained, which supports the idea that the effect of FCCP is related to mitochondrial inhibition.

The data above indicate that the metabolic inhibition evoked by FCCP induces a biphasic phenomenon, i.e., transient initial prolongation followed by a decrease in APD in Langendorff-perfused rabbit hearts.

### Effect of FCCP on APD Transmurally and Spatially in the Left Ventricle

We evaluated if the tendency for the biphasic effect of FCCP on the APD persisted transmurally and/or spatially. Pairs of glass microelectrodes were used to obtain paired AP recordings simultaneously at nearly the same place but at different depths, i.e., in the subepicardium and in subendocardium. Simultaneous recordings of both microelectrode – APs and OAPs enabled us to evaluate the differences in electrical APs depending on transmural and apical/basal heterogeneity resulting from different characteristics or expression levels of proteins involved in the ionic channels (Antzelevitch et al., 1991; Cheng et al., 1999; Lu et al., 2001) and depending on the location of cells in the heart. Previous studies have demonstrated that isolated endocardial and epicardial cardiomyocytes of the LV have different responses to metabolic inhibition under simulated ischemia (Khokhlova et al., 2017). Therefore, we also tested whether are the distinctions in the biphasic response of APD to metabolic inhibition evoked by FCCP in relation to the transmural or apical/basal location of cells in the LV wall based on measurements of the entire heart.

**FIGURE 2:**
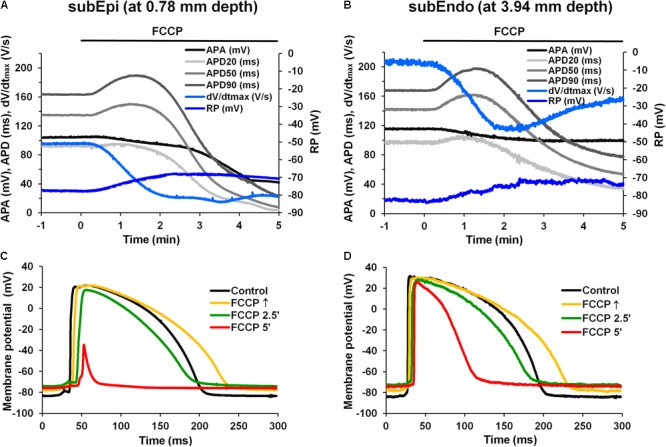
Effects of FCCP on subepicardial (subEpi) and subendocardial (subEndo) AP parameters in the left ventricles of rabbit hearts. **(A,B)** Typical examples of time dependent changes in simultaneous recordings of AP parameters in subEpi and subEndo cell layers, respectively. **(C,D)** Superimposition of the APs under control conditions (*black*) and under the effects of FCCP obtained from the subepicardium and subendocardium, respectively. Note the marked reduction in APs at FCCP 5′ in subEpi cells [**(C)**, *red*]. Other notations are the same as in **Figure 1**.

**Figure 2** compares the results of simultaneous recordings of APs from microelectrodes in subEpi and subEndo cardiomyocytes. One can see that the biphasic profile during perfusion with FCCP was characteristic for both subEpi, obtained at ∼0.8 mm depth, and subEndo, obtained at ∼4 mm depth, cardiomyocytes (**Figure 2A** vs. **Figure 2B**). At both cell layers, the APD evaluated at 20, 50, and 90% of repolarization (APD20, APD50, and APD90, respectively, in **Figures 2A,B**) significantly lengthened over time and reached its maximum prolongation between 30 and 90 s after FCCP treatment, which was followed by a significant shortening. Of note, the APA, dV/dt_max_, and RP were lower for subEpi than for subEndo cardiomyocytes (see also **Table 2**), in accordance with previous observations (Gilmour and Zipes, 1980).

**Table 2:** Changes of AP parameters in subepicardial (subEpi) and subendocardial (subEndo) cardiomyocytes under the effects of FCCP (*n* = 9 for each).

	dV/dt_max_ (V/s)	RP (mV)	APA (mV)	APD20 (ms)	APD50 (ms)	APD90 (ms)
subEpi						
Control	104.8 ± 6.7#	–76.1 ± 0.5#	104.3 ± 1.5#	85.3 ± 2.7	128.1 ± 2.4#	157.2 ± 2.1#
FCCP↑	69.0 ± 5.2*,#	–70.4 ± 1.4*,#	89.5 ± 5.2*	87.6 ± 2.8*	137.3 ± 2.9*	173.9 ± 3.4*
FCCP 2.5′	48.7 ± 5.4*,#	–67.5 ± 1.8*	87.9 ± 3.3*	58.9 ± 5.5*	92.4 ± 8.2*	122.9 ± 9.5*
FCCP 5′	49.1 ± 7.3*,#	–67.9 ± 1.4*	71.2 ± 7.9*	23.4 ± 5.7*	36.7 ± 8.3*	60.8 ± 9.4*
subEndo						
Control	148.5 ± 15.8	–79.1 ± 0.3	109.8 ± 1.5	87.6 ± 3.1	133.3 ± 2.3	161.3 ± 2.2
FCCP↑	107.7 ± 10.8*	–74.8 ± 0.9*	98.0 ± 3.9*	93.2 ± 2.8*	147.3 ± 3.5*	182.1 ± 3.8*
FCCP 2.5′	82.1 ± 9.7*	–70.9 ± 1.4*	89.3 ± 5.6*	62.8 ± 3.9*	98.1 ± 6.9*	127.4 ± 8.4*
FCCP 5′	92.0 ± 14.7*	–70.0 ± 2.4*	78.4 ± 9.4*	35.8 ± 6.1*	56.9 ± 8.5*	86.5 ± 7.7*

*Note that the depths of microelectrode insertion for subEpi and subEndo recordings were 0.59 ± 0.12 and 2.96 ± 0.18 mm from the epicardium, respectively. Other notations are the same as in **Table 1**. ^∗^*p* < 0.05 vs. control. ^#^*p* < 0.05 subEpi vs. subEndo values obtained at control and at the same time of FCCP action.*

**Figures 2C,D** shows the overlap of APs for the control condition and after different times of the treatment with FCCP, which were obtained from the LVs of rabbit hearts for two types of cells, subEpi and subEndo cardiomyocytes, respectively. In agreement with the data presented above, FCCP elicited an initial prolongation in both cell types (see **Figure 2C** vs. **Figure 2D**, *yellow*). However, after 5 min, much greater reductions in the magnitudes of the RP, APA, and APD were obtained in the subepicardium. These observations were also in agreement with other investigations showing that epicardial cells are more sensitive to metabolic inhibition (Gilmour and Zipes, 1980).

The mean values of AP parameters and the percentage changes in APDs were calculated from nine experiments in both cell types (subEpi vs. subEndo cardiomyocytes) after FCCP exposure and are presented in **Tables 2, 3**. Evaluation of the percentage changes in APDs in subEpi and subEndo cells revealed that the initial prolongation induced by FCCP at APD20 and at APD50 was more pronounced in subEndo than in subEpi cells. In addition, the shortening of APs detected after 5 min of FCCP treatment in both subEpi and subEndo cell layers was considerable (see **Table 3**).

**Table 3:** Percentage changes in APDs in subEpi and subEndo cardiomyocytes under the effects of FCCP (*n* = 9 for each).

	APD20 (%)	APD50 (%)	APD90 (%)
subEpi			
FCCP↑	2.8 ± 0.6#,*	7.1 ± 0.6#,*	10.5 ± 0.9*
FCCP 2.5′	–31.3 ± 5.6*	–28.4 ± 5.7*	–22.3 ± 5.3*
FCCP 5′	–72.1 ± 6.9*	–71.2 ± 6.5*	–61.3 ± 5.9*
subEndo			
FCCP↑	6.6 ± 1.1*	10.4 ± 0.9*	12.8 ± 1.1*
FCCP 2.5′	–28.8 ± 2.5*	–26.9 ± 4.1*	–21.5 ± 4.2*
FCCP 5′	–59.4 ± 6.5*	–57.5 ± 6.1*	–46.5 ± 4.5*

*Notations are the same as in **Tables 1, 2**. ^∗^*p* < 0.05 vs. control. ^#^*p* < 0.05 subEpi vs. subEndo values obtained at the same time of FCCP action.*

We also tested, whether a biphasic profile of the effects of FCCP also exists spatially in the apex-to-base direction. As presented in **Supplementary Figure S2**, the OAPD map obtained before FCCP application was fairly uniform throughout the mapping area for values calculated at 20% of repolarization (OAPD20), while at OAPD50 and OAPD90, values were slightly larger (∼5%) near the apex than at the base of the LV (**Supplementary Figure S2A**). Pretreatment with glibenclamide did not change the profile of the OAPD distribution (not shown). However, it is clear that apex-to-base heterogeneity of OAPD remained during treatment with FCCP (**Supplementary Figures S2B,C**). Such data were obtained in all four tested hearts.

**FIGURE 3:**
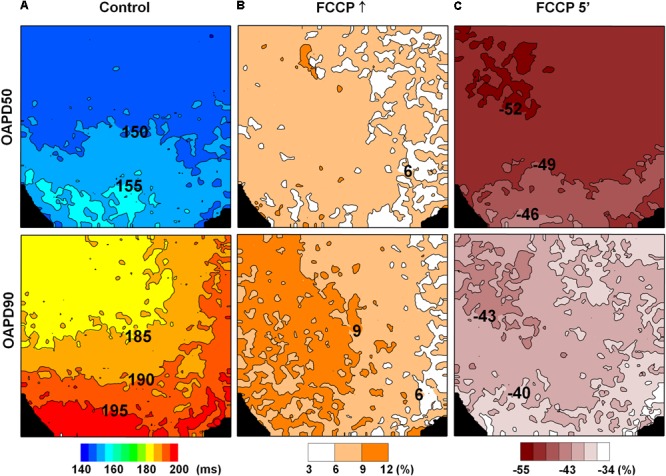
Changes in OAPD induced by FCCP after eliminating the impact of APD apical/basal heterogeneity. OAPD50 (*top row*) and OAPD90 (*bottom row*) maps obtained under control conditions [**(A)**, in ms] and normalized vs. control at the FCCP↑ and at 5 min of FCCP treatment, respectively **(B,C)**. Positive and negative percentages of OAPD changes show prolongation and shortening of the APs with respect to the control level. The corresponding scale-bars are given at the bottom.

To check if the effects of FCCP in inducing a biphasic profile were of the same magnitude in the apex-to-base direction, changes in OAPD detected during FCCP treatment were normalized vs. control values (without FCCP). **Figure 3** shows the maps demonstrating the actual impact of FCCP on APD, i.e., prolongation and shortening of OAPs, calculated as a difference in the corresponding OAPD50 and OAPD90 maps with respect to the control. Note that the OAPD maps for the control condition are presented in ms (**Figure 3A**). The results show that an initial increasing effect of FCCP on OAPD was fairly uniform, and OAPD was prolonged to a similar extent in both directions (**Figure 3B**). However, after 5 min of FCCP treatment, a decreasing effect was elicited, involving OAP shortening that was slightly heterogeneous. The OAP shortening was more pronounced moving from the apex of the LV toward the base (**Figure 3C**); this might indicate that the decreasing effect of the FCCP depends on differences in the dominant ion currents in the apical/basal direction. In the given example, such a distribution is more pronounced for OAPD50.

In summary, in entire isolated hearts, a biphasic response to metabolic inhibition likely does not depend on the transmural and/or spatial heterogeneity of cardiomyocyte location as FCCP induced a transient prolongation followed by a subsequent shortening of APs at each cell layer, both spatially and transmurally. It also should be noted that the magnitude of the prolongating effect of FCCP on APs in spatial/transmural areas of the heart was different.

### The Role of the Inward Currents in AP Prolongation

Considering the information presented above, it is likely that inward and/or outward ion currents may be involved in the biphasic response of APD to metabolic inhibition.

**FIGURE 4:**
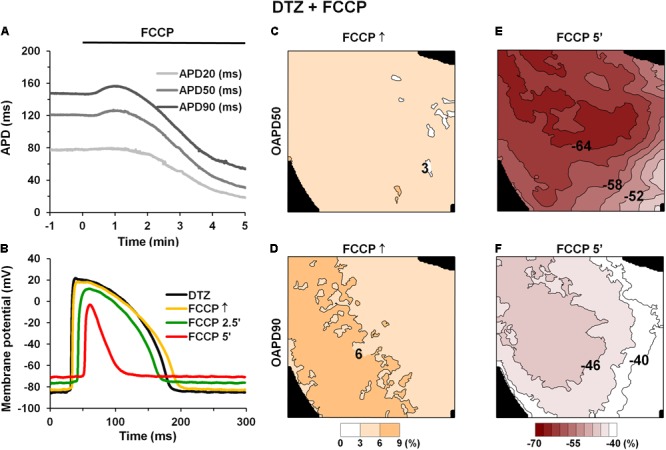
Effect of FCCP on APD after pretreatment with 10 μmol/L diltiazem. **(A)** Time-dependent changes in microelectrode-recorded APDs (APD20 – light gray, APD50 – gray, and APD90 – dark gray). FCCP perfusion started at time 0. **(B)** Superimposition of APs under the effects of FCCP in the presence of DTZ. **(C–F)** Simultaneously obtained OAPD maps at 50% (*top row*) and 90% (*bottom row*) of repolarization. OAPDs obtained at FCCP↑ **(C,D)** and at 5 min of FCCP treatment **(E,F)** and normalized vs. control. Numbers near isolines and scale bars (*bottom*) show OAPD changes in %. The interval between isolines on the maps is 3%. Other notations are the same as in **Figure 1**.

First, we tested if inward ion currents, e.g., an L-type calcium current (*I*_CaL_), may have impacts on the initial AP prolongation during FCCP treatment. For this test, the *I*_CaL_ antagonist DTZ (10 μmol/L) was used in some experiments. As is seen in **Figures 4A,B**, pretreatment with this drug did not abolish the biphasic profile of the metabolic inhibition induced by FCCP. DTZ by itself shortened APs at all repolarization levels (*n* = 4; *p* < 0.05 vs. control). The mean values for the effects of FCCP on the APD in the hearts pretreated with DTZ are shown in **Table 4**. Importantly, AP prolongation was recorded only at 50 and 90% of repolarization, and the magnitude of prolongation with DTZ was smaller than that without DTZ.

**Table 4:** APD changes after FCCP application in the presence of the various inhibitors.

	APD20 (%)	APD50 (%)	APD90 (%)
Control (*n* = 18)			
FCCP↑	4.7 ± 0.8*	8.8 ± 0.7*	11.7 ± 0.8*
FCCP 2.5′	–30.1 ± 2.9*	–27.6 ± 3.4*	–21.9 ± 3.3*
FCCP 5′	–65.7 ± 4.9*	–64.3 ± 4.6*	–53.9 ± 4.0*
DTZ (*n* = 4)	–10.9 ± 1.7#	–11.4 ± 0.5#	–9.7 ± 0.3#
DTZ + FCCP↑	–0.4 ± 0.8^†^	3.1 ± 0.8*^†^	5.2 ± 0.6*^†^
styDTZ + FCCP 2.5′	–21.3 ± 1.4*	–19.6 ± 0.9*	–17.2 ± 0.9*
DTZ + FCCP 5′	–71.8 ± 1.6*	–71.0 ± 1.8*	–61.7 ± 2.6*
Ranolazine (*n* = 4)	0.6 ± 1.6	1.1 ± 0.5#	2.3 ± 0.3#
Ran + FCCP↑	5.7 ± 1.6*	8.6 ± 0.8*	10.1 ± 0.8*
Ran + FCCP 2.5′	–29.3 ± 6.9*	–31.8 ± 7.2*	–26.3 ± 6.1*
Ran + FCCP 5′	–63.0 ± 9.3*	–63.0 ± 9.7*	–54.6 ± 9.9*
4-AP (*n* = 8)	20.8 ± 2.3#	20.3 ± 2.1#	17.0 ± 1.3#
4-AP + FCCP↑	3.8 ± 0.8*	7.3 ± 1.0*	9.1 ± 1.0*^†^
4-AP + FCCP 2.5′	–41.9 ± 5.4*	–43.1 ± 4.8*^†^	–36.0 ± 4.1*^†^
4-AP + FCCP 5′	–69.3 ± 5.1*	–71.2 ± 4.9*	–66.1 ± 5.0*
Oligomycin (*n* = 4)	3.3 ± 1.4	2.3 ± 1.2	2.1 ± 1.2
Oligo + FCCP↑	0.8 ± 0. 3^†^	1.7 ± 0.4^†^	2.9 ± 0.3^†^
Oligo + FCCP 2.5′	–1.8 ± 1.3^†^	–0.3 ± 0.3^†^	1.8 ± 0.2^†^
Oligo + FCCP 5′	–13.4 ± 3.1^†^	–9.6 ± 1.2^†^	–5.2 ± 0.5^†^

*Diltiazem (DTZ, 10 μmol/L) – L-type calcium channel blocker, ranolazine (Ran, 10 μmol/L) – late sodium channel blocker, 4-aminopyridine (4-AP, 2 mmol/L) – a transient outward K^+^ channel blocker, oligomycin A (Oligo, 30 μmol/L) – a mitochondrial F_*1*_F_*0*_ATPase inhibitor. The positive and negative percentages show prolongation and shortening of APs, respectively. Other notations are the same as in **Table 1**. ^∗^*p* < 0.05 with FCCP vs. before FCCP treatment. ^#^*p* < 0.05 drug (DTZ, Ran, 4-AP, Oligo) vs. control (see **Table 1**). ^†^*p* < 0.05 drug+FCCP vs. FCCP alone values obtained at the same time of FCCP action.*

The maps of OAPD50 and OAPD90 percent changes at FCCP↑ and after 5 min of FCCP treatment in the presence of DTZ are presented in **Figures 4C–F**, respectively. Notable, the interval between isolines (3%) was kept the same as in **Figure 3** in order to compare the changes under the effects of FCCP alone. The maps show that the apical/basal profile of OAPD changes in hearts under pretreatment with DTZ is similar to the effects of FCCP alone. Quantitative comparison revealed that FCCP in the presence of the *I*_CaL_ blocker evoked less prolongation of OAP at FCCP↑ (**Figures 4C,D**) compared with the effects of FCCP alone. However, OAP shortening after 5 min of FCCP treatment was more pronounced, especially for OAPD50.

The fact that FCCP resulted in greater prolongation of APs without DTZ pretreatment suggests that the biphasic effect might be related to the *I*_CaL_ but that it is not dependent only on this current. The cytosolic Ca^2+^ concentration and pH_i_, which change during metabolic inhibition, could participate in AP prolongation by exerting a slowing effect on the inactivation of the *I*_CaL_ (Sham et al., 1995; Saegusa et al., 2011).

**FIGURE 5:**
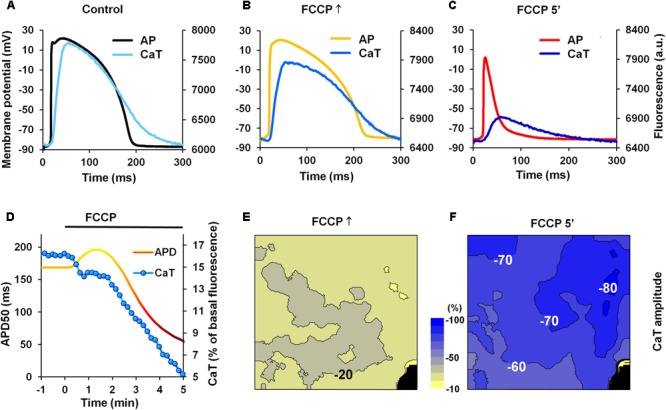
Effects of FCCP on CaTs. **(A–C)** Superimposition of simultaneously recorded microelectrode APs and CaTs obtained under control conditions and under the effects of FCCP. **(D)** A typical example of time-dependent changes in APD50 (*yellow-red*) and the amplitude of CaTs (*blue*). **(E,F)** The maps of changes in CaT amplitude at FCCP↑ and 5 min of FCCP treatment, respectively. Numbers near isolines and scale bar (*between CaT maps*) show the CaT amplitude changes in %. The interval between isolines on the maps is 10%. Negative values show the percent decrease of CaT amplitude from the control level. Note that decreases in CaT amplitude are greater as colors shift from yellow to blue. Other notations are the same as in **Figure 1**.

One of the reasons for the slowdown of *I*_CaL_ inactivation may be a decrease in CaTs; therefore, we performed a set of experiments in which the effect of FCCP on CaTs was investigated. Simultaneously, electrical AP measurements were performed. **Figures 5A–C** shows a typical example of microelectrode-recorded APs and CaTs under control conditions and under the effects of FCCP. The time-dependent changes in CaT amplitude and APD50 are presented in **Figure 5D**. CaTs decreased over time during metabolic inhibition, and a delay of the decrease in CaTs was observed that coincided with the time when AP prolongation was registered. This may be due to a greater inflow of Ca^2+^ ions through the *I*_CaL_ channels due to a prolonged AP causing an increase in a Ca^2+^-induced Ca^2+^ release. Further, steep decreases in both CaT amplitude and APD50 were observed. The maps presented in **Figures 5E,F** shows that the decline in CaT amplitude under the effects of FCCP occurred throughout the entire mapped LV surface. Of note, in order to eliminate the impact of possible illumination disparities, the amplitude of the signal was normalized with respect to the control level. After 5 min of FCCP treatment, the CaT amplitude was reduced by 76 ± 12% (*n* = 5).

Thus, simultaneous measurements of CaTs and AP show that CaTs are decreased when AP is prolonged. Reduced CaT can result in the slowdown of *I*_CaL_ channel inactivation and may be responsible, at least in part, for the prolongation of APs.

One of the consequences of metabolic inhibition is the decrease of intracellular pH (Carmeliet, 1999). This acidification changes the characteristics of various intracellular systems, including ion currents that form APs. The changes of AP parameters induced by intracellular acidification evoked by perfusion with sodium acetate are presented in **Supplementary Figure S3** and **Table S1**. The decreased pH_i_ impacts the dynamics of *I*_CaL_ inactivation by slowing it down, which results in AP prolongation (Saegusa et al., 2011). Therefore, we also measured pH_i_ changes during FCCP treatment using the SNARF-1 fluorescent dye. A decrease in pH_i_, which indicates intracellular acidification, was observed under the effects of FCCP. The results from simultaneous recordings of pH_i_ and APs are presented in **Supplementary Figure S4**. Again, APD changed in a biphasic manner, while pH_i_ declined immediately from the beginning of FCCP perfusion. The small decrease in pH_i_ and its subsequent partial recovery, starting at ∼3 min of FCCP treatment, could be explained by the activity of the sarcolemmal acid-transporting systems, such as Na^+^/H^+^ exchangers, Na^+^/${\mathrm{HCO}}_{3}^{–}$ cotransporters, and monocarboxylic acid transporters (Vaughan-Jones et al., 2009; Saegusa et al., 2011). Hence, our data are in accordance with other observations, suggesting that AP prolongation may be related to the delay of *I*_CaL_ inactivation due to intracellular acidification (Saegusa et al., 2011).

**FIGURE 6:**
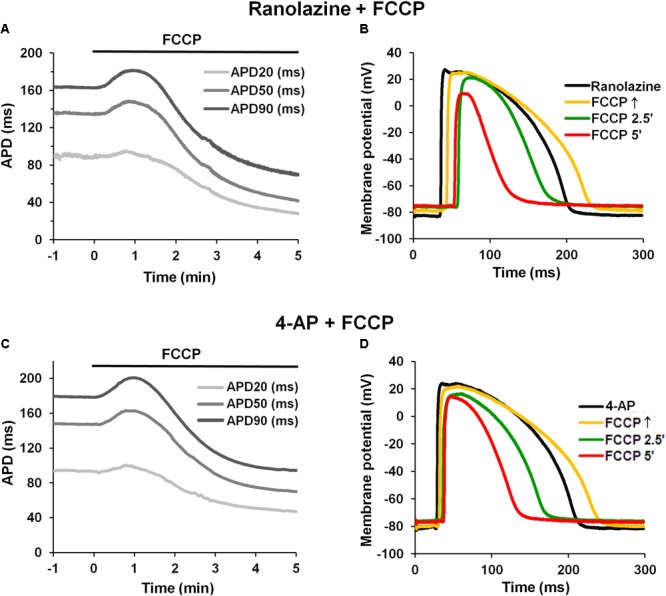
Effect of FCCP on APD after pretreatment with ranolazine or 4-AP. **(A,C)** Typical examples of time-dependent changes in microelectrode-recorded APs: APD20 (*light gray*), APD50 (*gray*), and APD90 (*dark gray*) after pretreatment with ranolazine (10 μmol/L) or 4-AP (2 mmol/L), respectively. FCCP perfusion started at time 0. **(B,D)** Superimposition of APs in the presence of ranolazine or 4-AP alone, respectively (*black*), and together with FCCP: at FCCP↑ (*yellow*), at 2.5 min (*green*), and at 5 min (*red*).

In addition, we tested whether the late sodium current (*I*_NaLate_), which likely increases during ischemic conditions (Ju et al., 1996; Saint, 2006), could be involved in APD changes upon metabolic inhibition. **Figures 6A,B** shows typical records of APD changes at different times during perfusion with FCCP in hearts pretreated with the *I*_NaLate_ blocker ranolazine. Ranolazine (10 μmol/L) by itself slightly prolonged APs (see **Table 4**). The subsequent application of FCCP in the presence of ranolazine did not eliminate the biphasic profile of APD.

These results indicate that the *I*_CaL_ might participate in AP prolongation, whereas the inward *I*_NaLate_ may not be involved in the initial prolongation of APs during metabolic inhibition.

### The Role of Outward Currents on AP Prolongation

There are no data showing that metabolic inhibition can reduce the main repolarizing currents *I*_Kr_ and *I*_Ks_. *I*_Kr_ may be reduced during ischemia, but exclusively due to external acidosis (Carmeliet, 1999). Furthermore, the recent experimental studies with single guinea pig cardiomyocytes showed that *I*_Kr_ decreases during reperfusion after ischemia but remains unchanged during chemical anoxia (Chen et al., 2017). In our experiments, the pH of the perfusion solution was kept constant at physiological level. Notwithstanding, we have performed experiments in which the effect of FCCP was tested in the presence of the *I*_Kr_ and *I*_Ks_ blockers E-4031 and HMR-1556, respectively. As expected, the block of *I*_Kr_ and *I*_Ks_ did not eliminate the AP prolongation induced by FCCP (see **Supplementary Figure S5**).

Furthermore, we tested whether the transient outward K^+^ current (*I*_to_), which decreases during the inhibition of oxidative phosphorylation (Ogbaghebriel and Shrier, 1994; Barth and Tomaselli, 2009), could be involved in AP prolongation. **Figures 6C,D** shows typical records of APD changes during perfusion with FCCP in hearts pretreated with 2 mmol/L 4-AP, a blocker of *I*_to_. It is obvious that 4-AP did not eliminate the FCCP-induced AP prolongation. 4-AP by itself significantly (*p* < 0.05 vs. control) prolonged APs at APD20, APD50, and APD90. The mean values for the effects of FCCP on APD in hearts pretreated with 4-AP are shown in **Table 4**. Of note, the subsequent application of FCCP in the presence of 4-AP did not eliminate the biphasic profile of APD changes over time. However, the results of 4-AP treatment presented in **Table 4** are given irrespective of the transmural dispersion of the *I*_to_. In the rabbit heart, the density of *I*_to_ channels in the epicardium of the LV is higher than that in the endocardium (Fedida and Giles, 1991); therefore, to determine the possible differences between both cell layers, we separated subEpi APs from subEndo APs, and these data are shown in **Table 5**. As expected, 4-AP itself prolonged APs in subEpi cells to a greater extent than in subEndo cells. Under the effects of FCCP and in the presence of 4-AP, when the *I*_to_ is almost blocked, AP prolongation was still recorded in both cell layers, but to a significantly lesser degree in subEpi cells than in subEndo cells. Compared with the results of FCCP treatment only (see **Table 3**), FCCP in the presence of 4-AP prolonged APs to a lesser extent in both subEpi and subEndo cells, but significantly only for subEpi cells.

**Table 5:** Percentage changes in APD in subEpi and subEndo cardiomyocytes after FCCP application in addition to 4-AP (2 mmol/L) (*n* = 4 for each).

	APD20 (%)	APD50 (%)	APD90 (%)
subEpi			
4-AP	26.3 ± 1.2#,*	24.0 ± 2.0#,*	19.2 ± 1.3#,*
FCCP↑	2.2 ± 0.9#	4.4 ± 0.4#,*	6.3 ± 0.1#,*
FCCP 2.5′	–56.9 ± 1.8#,*	–56.4 ± 1.5#,*	–47.6 ± 1.1#,*
FCCP 5′	–83.3 ± 0.9#	–84.4 ± 0.6#	–79.7 ± 0.4#
subEndo			
4-AP	15.3 ± 1.9*	16.7 ± 2.6*	14.8 ± 1.8*
FCCP↑	5.5 ± 0.6	10.1 ± 0.1	11.9 ± 0.1
FCCP 2.5′	–26.9 ± 0.8	–29.8 ± 1.2	–24.5 ± 0.4
FCCP 5′	–55.3 ± 2.6	–58.1 ± 2.7	–52.4 ± 2.6

*Note that the depths of microelectrode insertion for subEpi and subEndo recordings were 0.48 ± 0.3 and 3.1 ± 0.2 mm from the epicardium, respectively. The positive/negative percentages show the prolongation/shortening of APs, respectively. Other notations are the same as in **Table 1**. ^∗^*p* < 0.05 with 4-AP vs. without 4-AP (see **Table 3**) values obtained at the same time of FCCP action. ^#^*p* < 0.05 subEpi vs. subEndo values obtained with 4-AP and at the same time of FCCP action.*

Our data confirm the unequal distribution of *I*_to_ channels in both layers (because of the greater prolongation of APs in subEpi vs. subEndo cells under 4-AP treatment) and coincide with the data reported by Fedida and Giles (1991), which showed that the density of *I*_to_ channels in SubEndo cells isolated from rabbit heart is ∼30% less than that in SubEpi cells. However, the fact that under conditions when the *I*_to_ is almost blocked, the observed differences in AP prolongation caused by FCCP in subEpi and subEndo cells may be explained by an unequal distribution or sensitivity to metabolic inhibition of other ion channels that participate in AP prolongation.

In summary, our results suggest that the *I*_to_ participates in the initial prolongation of AP during FCCP treatment.

**FIGURE 7:**
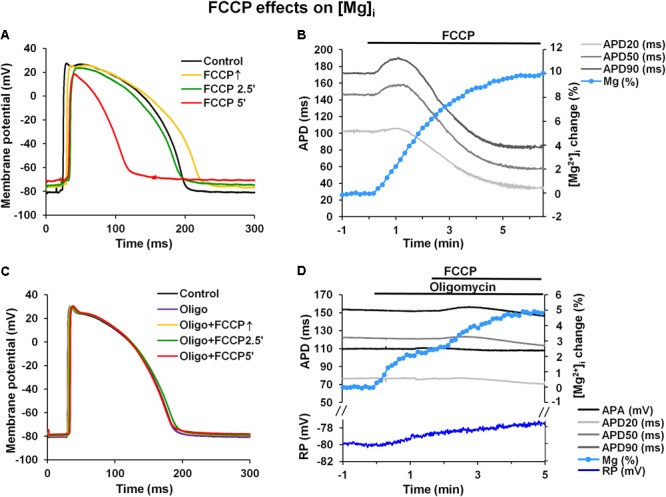
FCCP induces an increase in intracellular magnesium levels. **(A,C)** Superimposition of APs under control conditions (*black*) and at FCCP↑ (*yellow*), at 2.5 min (*green*), and at 5 min (*red*) of FCCP treatment before and after pretreatment with oligomycin (30 μmol/L), respectively. **(B,D)** Simultaneous records of time-dependent fluorescence changes showing changes in intracellular Mg^2+^ concentration ([Mg^2+^]_i_, *light blue*), resting membrane potential [*blue*, only in **(D)**], and microelectrode-recorded AP duration: APD20 (*light gray*), APD50 (*gray*), and APD90 (*dark gray*) before and after pretreatment with oligomycin, respectively. The change in [Mg^2+^]_i_ is presented in the % of the control level.

The results presented above (see **Figure 1** and **Table 1**) clearly show that FCCP causes cell membrane depolarization. Since the inward rectifying potassium current (*I*_K1_) is the main current responsible for maintaining the RP it is obvious that this current is affected during metabolic inhibition. Previously, it has been shown that there are no differences in the magnitude of the *I*_K1_ in subEpi and subEndo cells (Liu et al., 1993) and that this current is sensitive to increased [Mg^2+^]_i_ (Matsuda et al., 1987; Carmeliet, 1999). Under metabolic inhibition [Mg^2+^]_i_ may increase, and this could be a reason for the decrease in the *I*_K1_, which in turn could result in the prolongation of APs and depolarization of the RP. To determine if the *I*_K1_ may be involved in AP prolongation (Warren et al., 2003; Hoeker et al., 2017), changes in [Mg^2+^]_i_ were measured under the effects of FCCP. **Figures 7A,B** shows typical examples of microelectrode-recorded APs (**Figure 7A**) and simultaneously recorded time-dependent changes in APD and [Mg^2+^]_i_ (**Figure 7B**). The data show that under the effects of FCCP, [Mg^2+^]_i_ gradually increased. After 5 min of FCCP treatment, the level of [Mg^2+^]_i_ had increased by 11.18 ± 1.70% (*n* = 6) relative to the control level.

An increased [Mg^2+^]_i_ has also been recorded in various types of single cells under metabolic inhibition (Leyssens et al., 1996; Kubota et al., 2005; Zima et al., 2013). However, the values obtained in single cells are higher than those found in our study, which could be explained, at least in part, by an inner filter effect (Kao et al., 1998), particular as a result of the self-shielding (which is more extensive in the entire heart than in single cells) that occurs when the dye itself attenuates the penetration of the excitation light to the deeper myocardial layers and attenuates the emission of the fluorescence from these layers. Since ATP has a high affinity for Mg^2+^, the major portion of the intracellular store of Mg^2+^ is found as MgATP (Leyssens et al., 1996; Romani, 2011). ATP hydrolysis during metabolic inhibition induces an increase in [Mg^2+^]_i_; therefore, it is generally accepted that changes in [Mg^2+^]_i_ directly reflect intracellular changes in ATP during metabolic inhibition (Leyssens et al., 1996; Romani, 2011).

The use of mitochondrial uncouplers, which diminish the mitochondrial membrane potential, undoubtedly block ATP synthesis and induce cytosolic ATP hydrolysis. Therefore, the ATP concentration decreases, [Mg^2+^]_i_ increases and there is intracellular acidification. Mitochondrial uncouplers, due to the reduced mitochondrial membrane potential itself, might also impact the balance of cytosolic electrolytes.

It is generally accepted that the main Mg^2+^ source during metabolic inhibition is ATP hydrolysis. In addition, it is known that mitochondria serve as an important source of Mg^2+^. Recently, it was demonstrated that after exposure to FCCP, the Mg^2+^ levels in cytosol increased, while at the same time, those in mitochondria decreased (Yamanaka et al., 2016). These data suggest that the origin of the increase in Mg^2+^ levels during the first minutes of metabolic inhibition could be due, at least in part, to the release of Mg^2+^ from mitochondria. To evaluate the impact of mitochondrial Mg^2+^ on the increase of cytosolic Mg^2+^ upon the effect of FCCP, we performed experiments with oligomycin, a mitochondrial F_1_F_0_ATPase inhibitor, which blocks ATP synthesis and abolishes cytosolic ATP hydrolysis. **Figures 7C,D** illustrate a typical example of microelectrode-recorded APs (**Figure 7C**) and simultaneously recorded time-dependent changes in APD and [Mg^2+^]_i_ (**Figure 7D**) under the effects of FCCP in the presence of 30 μmol/L oligomycin. Oligomycin alone had no effect on APD or the RP but induced a slight increase in [Mg^2+^]_i_ (2.24 ± 0.06% (*n* = 4) of the control level). FCCP with oligomycin further increased [Mg^2+^]_i_ to 5.82 ± 0.64% (*n* = 4), but the increase was less than without oligomycin (*p* < 0.05). Moreover, the data show that in the presence of oligomycin, FCCP elicited much weaker biphasic effect on APD (**Table 4**), and depolarization of the RP was also reduced (*blue*, **Figure 7D**).

## Discussion

Our study provides the first spatio-transmural characterization of the evolution of the biphasic effect during metabolic inhibition in the Langendorff-perfused rabbit heart. We demonstrate that the APD significantly changes over time and has a biphasic character, reaching its maximum prolongation at approximately 1 min after FCCP application, followed by a significant shortening at 5 min. In addition, metabolic inhibition reduces RP, APA, and dV/dt_max_ and slows down AP conduction. It is known that oxygen consumption is greater in the endocardium (Beyar and Sideman, 1986); consequently, the vulnerability to ischemia is also higher. Nevertheless, electrophysiological changes, e.g., shortening of the AP, are greater in the subepicardium (Furukawa et al., 1991) due to the higher *I*_to_ current density (Antzelevitch et al., 1991), and the larger sensitivity of *I*_CaL_ (Kimura et al., 1991) and *I*_Na_ (Cordeiro et al., 2008) currents to ischemia in epicardial cells. We recorded AP prolongation in both subEpi and subEndo cells, but the effect was more pronounced in the subendocardium. It should be noted that our experiments were performed under *I*_KATP_ channel blockade by maintaining a stable concentration (10 μmol/L) of glibenclamide, which allowed the delay of AP shortening and avoided the early development of ventricular fibrillation upon FCCP administration (Smith et al., 2012).

It has been documented that metabolic inhibition elicited by the application of FCCP could cause various intracellular alterations, including acidification, a decrease in [ATP]_i_, an increase in free [Mg^2+^]_i_, changes in Na^+^, K^+^, and Ca^2+^ homeostasis, etc. Consequently, the activities of many ion channels and exchangers are changed (Carmeliet, 1999). In contrast to heart ischemia, when the coronary blood circulation is stopped, under our experimental conditions, extracellular electrolytes and metabolites did not accumulate. Disbalance of electrolytes and their accumulation during ischemia have an important secondary effects on electrophysiological parameters due to changes in ion channels and exchanger activities (Taylor et al., 2013).

Changes in the APD, i.e., prolongation and shortening, were documented under ischemic/hypoxic conditions and during reperfusion (Garg et al., 2015; Barbic et al., 2017; Chen et al., 2017; Garrott et al., 2017). The activation of ATP-dependent K^+^ channels due to a lack of ATP results in AP shortening (Benndorf et al., 1992). However, our experimental data show that FCCP treatment during the first minute of the effects of an uncoupler could increase APD, especially in cases when *I*_KATP_ is suppressed. The phenomenon of the initial prolongation of APD in the first minutes after metabolic inhibition evoked by DNP (2,4-dinitrophenol), a classic uncoupler of oxidative phosphorylation, was first described long ago (Carmeliet and Boulpaep, 1957; Carmeliet, 1978). Later, it was suggested that the inhibition of the electrogenic sodium pump caused by ATP depletion is the underlying mechanism of this transient effect of metabolic inhibition (Isenberg et al., 1983). A study on isolated rabbit and human ventricular myocytes showed that the decrease in the *I*_to_, which has different distributions among epicardial and endocardial myocytes, may be responsible for initial prolongation of APD during metabolic inhibition (Verkerk et al., 1996). To date, there have been many studies demonstrating the effects of metabolic inhibition or the effects of the consequence of this inhibition, such as ATP depletion and acidification, on the different ionic currents that form APs (Ju et al., 1996; Murphy et al., 2011; Saegusa et al., 2011; Zima et al., 2013). However, there is a lack of knowledge regarding the mechanism underlying this phenomenon in whole heart preparations and of how the transmural and apical/basal heterogeneity of ionic currents impacts the effects of metabolic inhibition.

Elongation of APs could be elicited by an increase in inward currents or by a decrease in outward currents (Carmeliet, 1999). Although almost all membrane currents change their activity during metabolic inhibition, only a few of them can be responsible for the prolongation of APs, with the main ones as follows: the late sodium current (*I*_NaLate_), which likely appears during metabolic injury (Ju et al., 1996; Saint, 2006); the L-type Ca^2+^-current (*I*_CaL_), the inactivation of which can be delayed by intracellular acidification (Saegusa et al., 2011), or a failure of SR function (Sham et al., 1995); the well-known metabolic inhibition of the *I*_to_, which is responsible for the initial repolarization of APs; and a reduction of the inward rectifier K^+^ current (*I*_K1_), which is responsible for the level of the resting potential and the final phase of AP repolarization. The *I*_K1_ can be reduced during metabolic inhibition due to an increase in the free Mg^2+^ concentration. We used blockers of different currents to test which of them were responsible for the initial metabolic prolongation of APs.

Our results show that the *I*_NaLate_ is unlikely to be responsible for the AP elongation caused by FCCP since pretreatment of the heart with ranolazine, a blocker of the *I*_NaLate_, did not eliminate this effect. Meanwhile, according to our results, the *I*_CaL_ may be partially responsible for this phenomenon since under pretreatment with diltiazem, a blocker of the *I*_CaL_, the prolongation of AP was less than under the effect of FCCP alone. The prolongation of APs due to the *I*_CaL_ can be explained by metabolic intracellular acidification or by a reduction in the CaTs in response to attenuated SR function.

To evaluate the possible effects of outward K^+^ currents on APD during metabolic inhibition, we first checked if the 4-AP-sensitive *I*_to_ may be involved. Since it was demonstrated that inhibition of oxidative phosphorylation by DNP or cyanide decreased the magnitude of the *I*_to_ (Ogbaghebriel and Shrier, 1994; Barth and Tomaselli, 2009), this may be the reason for AP prolongation. It was also demonstrated that there is a different distribution of *I*_to_ channels among myocardial cells: the *I*_to_ channels in rabbit ventricles are more pronounced in epicardial than in endocardial cells (Fedida and Giles, 1991; Verkerk et al., 1996). In our experimental conditions, the effect of 4-AP, a blocker of the *I*_to_, on APD was present in both subEpi and subEndo cells. Importantly, in contrast to the findings of a previous study (Verkerk et al., 1996), our results showed that pretreatment with high concentration (2 mmol/L) of 4-AP did not eliminate the AP prolongation evoked by FCCP. In addition, it should be noted that the AP prolongation was reduced in both cell layers, although the reduction was significant only in subEpi cells. The different experimental conditions might be the reason for such discrepancies. Our findings suggest that in our experimental conditions, the initial increase in APD of Langendorff-perfused rabbit hearts under metabolic inhibition could be partially due to a decrease in the *I*_to_.

In our study, during the uncoupling of mitochondrial oxidative phosphorylation, an increase in [Mg^2+^]_i_ was observed. Under these conditions, the ATP hydrolysis is considered to be the main Mg^2+^ source. However, in the experiments with the F_1_F_0_ATPase inhibitor oligomycin, when ATP synthesis and hydrolysis were blocked, the increase in Mg^2+^ was still observed, though at a lesser extent. It was proposed that mitochondrial Mg^2+^ could be an additional source for increased Mg^2+^ during metabolic inhibition (Kubota et al., 2005; Yamanaka et al., 2016). We can also suggest that an increase in [Mg^2+^]_i_ under the effect of FCCP is not only related to ATP hydrolysis but may also be due to an additional Mg^2+^ release from mitochondria due to mitochondrial depolarization. Nevertheless, increased [Mg^2+^]_i_ may be responsible for the reduction in *I*_K1_. The decrease in the *I*_K1_ may participate in AP prolongation and depolarization of the resting potential. Both effects were clearly observed in our study.

It worth mentioning that an ischemia-induced increase in [Mg^2+^]_i_ and the subsequent decrease in *I*_K1_ might have a physiological protective effect. Changes in APD can be either arrhythmic or antiarrhythmic. During ischemia, activation of the *I*_KATP_ dramatically shortens APs, and this efficiently decreases the utilization of energy resources and thereby protects against myocardial necrosis. However, the dramatic shortening of APs can facilitate circular arrhythmias. The additional ischemic inhibition of the *I*_K1_, which prolongs APs at the late phase of AP repolarization, may prevent the circulation of electrical signals and have an antiarrhythmic effect (Jalife, 2009).

Thus, our results showed that in the whole heart, the mechanism of AP prolongation during metabolic inhibition is multidimensional compared with observations at the single-cell level. Our data obtained in the whole Langendorff-perfused rabbit heart suggest that the main currents responsible for AP prolongation are the *I*_CaL_, *I*_to_, and *I*_K1_, the activities of which were modulated mainly by changes in intracellular ATP, calcium, magnesium, and pH.

### Limitations

Optical/electrical signals interfere with mechanical contractions; therefore, the heart tissue was immobilized using blebbistatin (Fedorov et al., 2007). The removal of mechanical function of the heart creates energy-preserving conditions; therefore, the results showing the effects of metabolic inhibition in our experiments might be less acute than those from a beating heart.

Action potential duration changes in our experiments were measured under partial blockade of *I*_KATP_ channels by glibenclamide. This has helped us to determine the mechanism of early AP prolongation evoked by FCCP more precisely, but at the same time partially distorted the exact dynamics of the APD change induced by metabolic inhibition.

We did not record APs from the actual endocardial surface; instead, we recorded APs from the subendocardium because the thickness of the LV wall could be measured only after the termination of the experiment.

The pH_i_ measurements in our experiments were performed on the whole heart, and changes in pH_i_ during FCCP treatment were small compared with data reported for single cardiomyocytes (Zima et al., 2013). This could be due to calibration problems. Under our experimental conditions, calibration was conducted on a homogenized heart. In addition, SNARF-1 was excited using a 505 nm LED, which detects signals from the epicardium but not from the deep layers of the heart. Therefore, under such circumstances, is impossible to present the actual values of the pH_i_, and we can only state that during FCCP treatment, pH_i_ was reduced.

We did not perform direct measurements of *I*_K1_ current involvement in AP prolongation induced by FCCP, and the statement on *I*_K1_ participation in AP prolongation was based on intracellular magnesium increase and resting potential depolarization during metabolic inhibition.

In accordance with the principles of animal welfare, the data for this study were collected using minimal numbers of animals, especially when changes in registered parameters were of the same tendency.

## Conclusion

In the whole heart during metabolic inhibition, APD significantly changes over time and has a biphasic character, reaching maximum prolongation after approximately 1 min of FCCP treatment, followed by marked shortening at 5 min. At the same time, there were no substantial transmural and apical/basal differences in the biphasic effect induced by FCCP.

The AP prolongation evoked during the first minute of FCCP treatment can be explained by increased activity of the *I*_CaL_ and decreased activity of the *I*_to_ and *I*_K1_, apparently due to changes in intracellular ATP, calcium, magnesium, and pH.

## Author Contributions

JJ designed the research. AN contributed reagents, materials, and analytical tools. RM, RT, and JJ performed the experiments. RM, IM, IA, and JJ analyzed the data and created the figures. RM, IM, and JJ wrote the article. RM and JJ are joint senior authors. All authors reviewed and approved the final version of the manuscript.

## Conflict of Interest Statement

The authors declare that the research was conducted in the absence of any commercial or financial relationships that could be construed as a potential conflict of interest.

## Abbreviations

AP: action potential
APA: action potential amplitude
APD: action potential duration
CaT: calcium transient
DTZ: diltiazem
dV/dt_max_: maximal value of the first time derivative of the AP upstroke
FCCP: carbonyl cyanide-*p*-trifluoromethoxyphenylhydrazone
FCCP↑: the time of the maximal prolongation of AP upon FCCP action
4-AP: 4-aminopyridine
*I*_CaL_: L-type calcium current
*I*_K1_: inward rectifier potassium current
*I*_KATP_: ATP-sensitive potassium current
*I*_Kr_: delayed rectifying fast potassium current
*I*_Ks_: delayed rectifying slow potassium current
*I*_NaLate_: late sodium current
*I*_to_: transient outward potassium current
LV: left ventricle
OAP: optical action potential
RP: resting membrane potential
SR: sarcoplasmic reticulum
subEndo: subendocardial
subEpi: subepicardial
