# Supplementary material for: Mechanism of Action Potential Prolongation During Metabolic Inhibition in the Whole Rabbit Heart
Source: Front Physiol. 2018 Aug 9;9:1077. doi: 10.3389/fphys.2018.01077 (PMC6095129; doi:10.3389/fphys.2018.01077)
Supplement: Supplementary file 1 [file Table_1.PDF]

Table S1

The mean values of the effects of Na^+-acetate (Acetate) on AP parameters (n = 3 for each).

	**dV/dt_{max} (V/s)**	**RP (mV)**	**APA (mV)**	**APD20 (ms)**	**APD50 (ms)**	**APD90 (ms)**
Control	127.3 ± 29.1	-77.2 ± 1.4	105.2 ± 2.0	91.3 ± 2.7	132.0 ± 1.5	159.9 ± 1.6
Acetate ↑	123.6 ± 29.4	-78.8 ± 1.8	107.1 ± 1.5	94.9 ± 2.2	138.7 ± 1.0*	167.8 ± 2.0*
Acetate 2.5'	111.6 ± 30.1	-79.6 ± 2.2	102.1 ± 3.3	63.1 ± 1.7*	95.6 ± 0.3*	126.6 ± 1.5*
Acetate 5'	107.7 ± 31.7	-76.0 ± 6.6	93.6 ± 7.4	53.3 ± 1.4*	84.0 ± 2.5*	114.6 ± 1.0*

Note that the calculated mean depth was of 2.97 ± 1.2 mm. Other notations are the same as in Table 1 (in the Manuscript).

*$p < 0.05$ acetate vs. control.
