# Supplementary material for: Mechanism of Action Potential Prolongation During Metabolic Inhibition in the Whole Rabbit Heart
Source: Front Physiol. 2018 Aug 9;9:1077. doi: 10.3389/fphys.2018.01077 (PMC6095129; doi:10.3389/fphys.2018.01077)
Supplement: Supplementary file 2 [file Image_1.PDF]

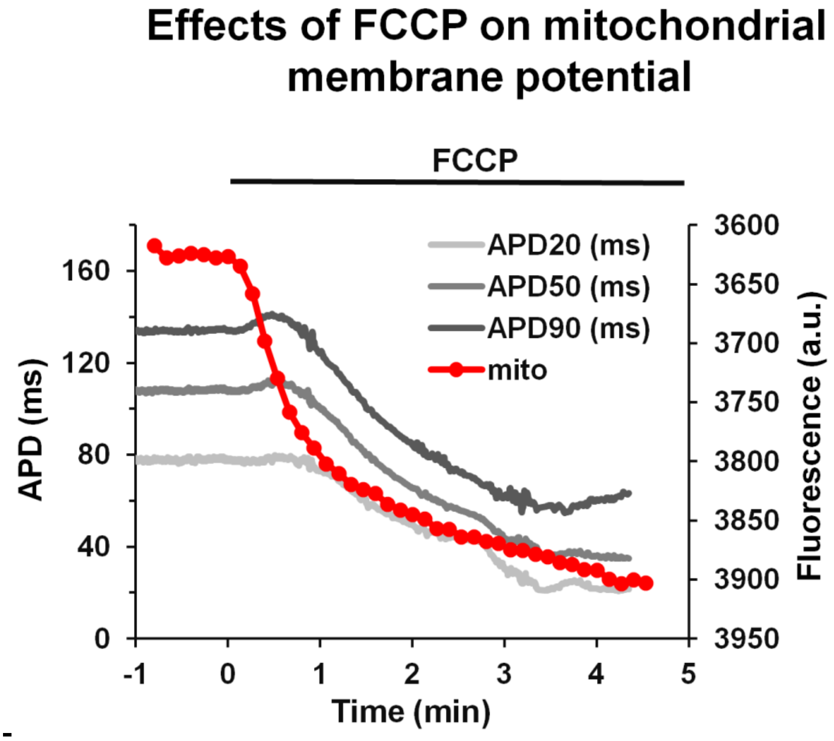

Figure S1

FCCP (1 µmol/L) induces mitochondrial uncoupling in Langendorff-perfused rabbit heart. Simultaneous recordings of time-dependent fluorescence changes showing alterations of intracellular mitochondrial membrane potential (mito, *red*) recorded with Mitotracker Deep Red (10 µmol/L) and changes in microelectrode-recorded AP durations: APD20 (*light grey*), APD50 (*grey*) and APD90 (*dark grey*). Note that FCCP perfusion starts at time zero.
