# Supplementary material for: Mechanism of Action Potential Prolongation During Metabolic Inhibition in the Whole Rabbit Heart
Source: Front Physiol. 2018 Aug 9;9:1077. doi: 10.3389/fphys.2018.01077 (PMC6095129; doi:10.3389/fphys.2018.01077)
Supplement: Supplementary file 3 [file Image_2.PDF]

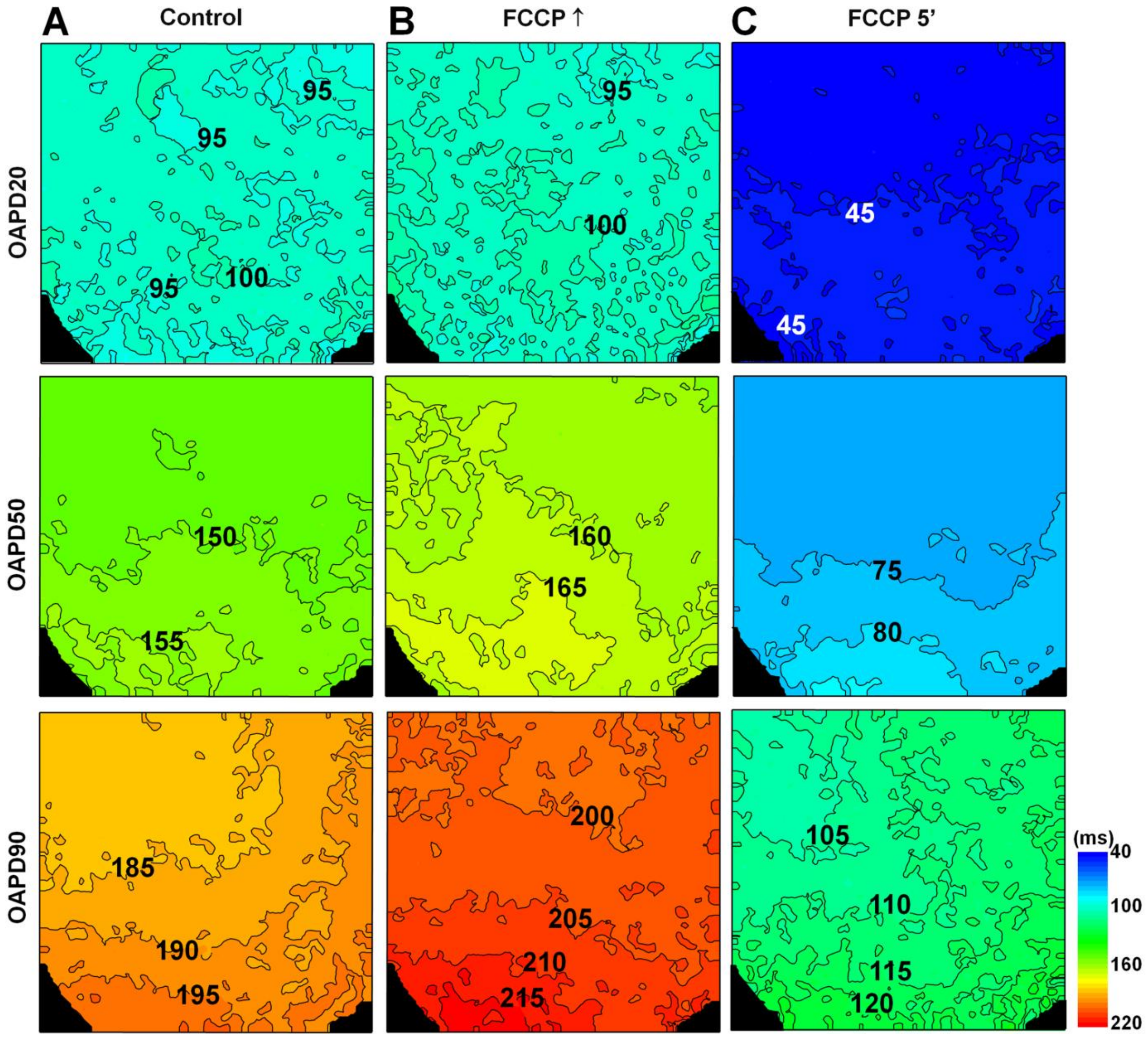

<u>Figure S2</u>

Representative OAPD maps obtained under control conditions vs. FCCP (1 µmol/L) treatment in Langendorff-perfused rabbit heart. OAPD maps obtained using the voltage-sensitive di-4-ANBDQBS dye at 20% (OAPD20, *top row*), at 50% (OAPD50, *middle row*), and at 90% (OAPD90, *bottom row*) of repolarization under control conditions **(A)** vs. at FCCP↑ and after 5 minutes of FCCP treatment, respectively **(B, C)**. OAPD maps were obtained from the same experiment as shown in Figure 1 (in the Manuscript). Numbers near isolines show the OAPD changes in ms. The interval between isolines on the maps is 5 ms.
