# Supplementary material for: Mechanism of Action Potential Prolongation During Metabolic Inhibition in the Whole Rabbit Heart
Source: Front Physiol. 2018 Aug 9;9:1077. doi: 10.3389/fphys.2018.01077 (PMC6095129; doi:10.3389/fphys.2018.01077)
Supplement: Supplementary file 4 [file Image_3.PDF]

Effect of acidification on APD changes

We applied sodium acetate (80 mM) in some experiments to induce intracellular acidification. Our data show (Figure S3A,B) that APD was increased during the first minutes of perfusion with sodium acetate. This effect is in accordance with other observation that intracellular acidification increases APD due to a delay of I_{CaL} inactivation (Saegusa et al., 2011). The initial prolongation of AP was followed by a decrease in APD, and this could be explained by the activity of sarcolemmal acid-transporting systems, such as Na^+/H^+ exchangers, Na^+-HCO_3^- cotransporters, and monocarboxylic acid transporters. The mean values for the effects of acidification on the AP parameters are shown in Table S1. The maps for OAPD50 and OAPD90 show that both were prolonged in the range of 0-6% (Figure S3C,D), whereas the decrease was more pronounced for OAPD50 (Figure S3E,F).

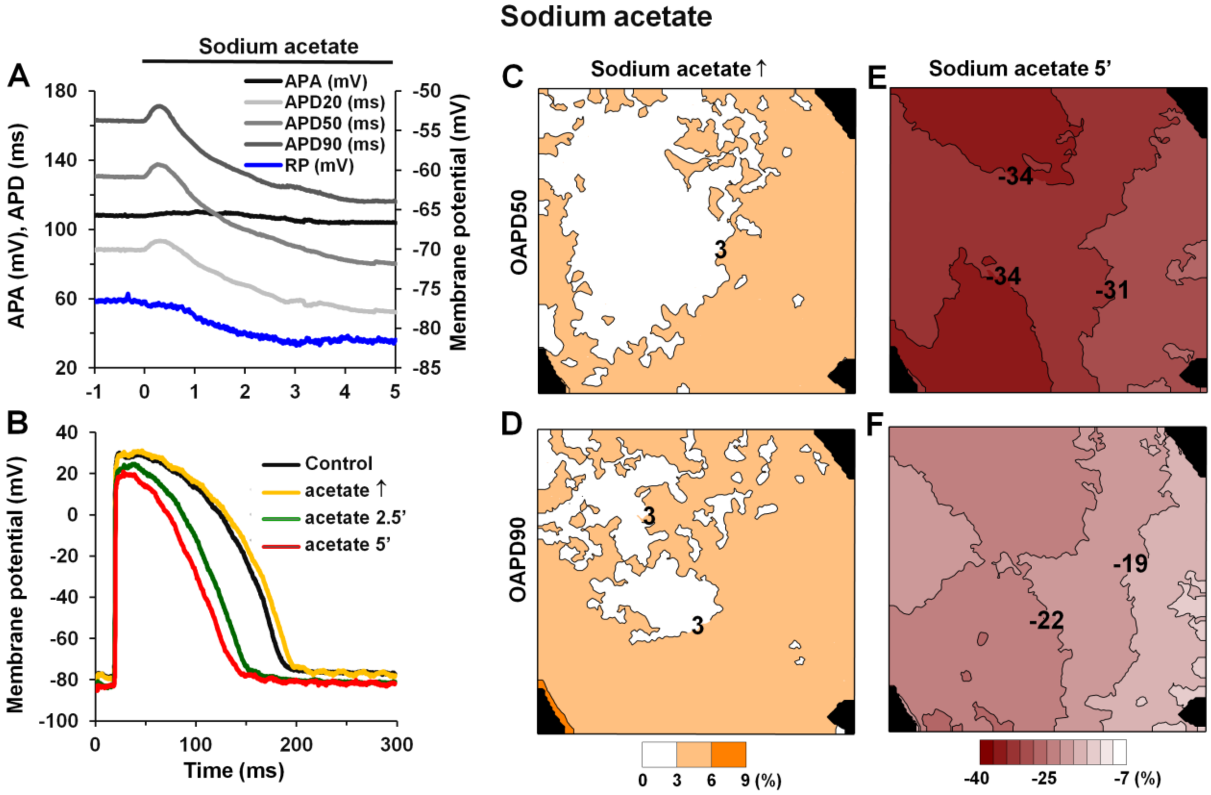

Figure S3

Effect of sodium acetate on APD and OAPD changes in Langendorff-perfused rabbit heart. **(A)** A typical example of time-dependent changes of microelectrode-recorded AP parameters: the amplitude (APA, *black*), the duration at 20% (APD20, *light grey*), 50% (APD50, *grey*) and 90% (APD90, *dark grey*) of repolarization, and the resting membrane potential (RP, *dark blue*). Sodium acetate perfusion started at time zero. **(B)** Superimposition of APs recorded under control conditions (*black*) and following acidification at the time of maximal AP prolongation (*yellow*), 2.5 (*green*) and 5 (*red*) minutes. Note the slight lengthening *vs.* shortening effects of acidification. **(C-F)** Simultaneously obtained OAPD maps, using the voltage-sensitive di-4-ANBDQBS dye at 50% and 90% of repolarization. Numbers near isolines and scale bar (*bottom*) show the OAPD changes in %. The interval between isolines on the maps is 3%. Note the magnitude of the OAPD changes increases as colours shift from white to orange/wine.
