# Supplementary material for: Mechanism of Action Potential Prolongation During Metabolic Inhibition in the Whole Rabbit Heart
Source: Front Physiol. 2018 Aug 9;9:1077. doi: 10.3389/fphys.2018.01077 (PMC6095129; doi:10.3389/fphys.2018.01077)
Supplement: Supplementary file 5 [file Image_4.PDF]

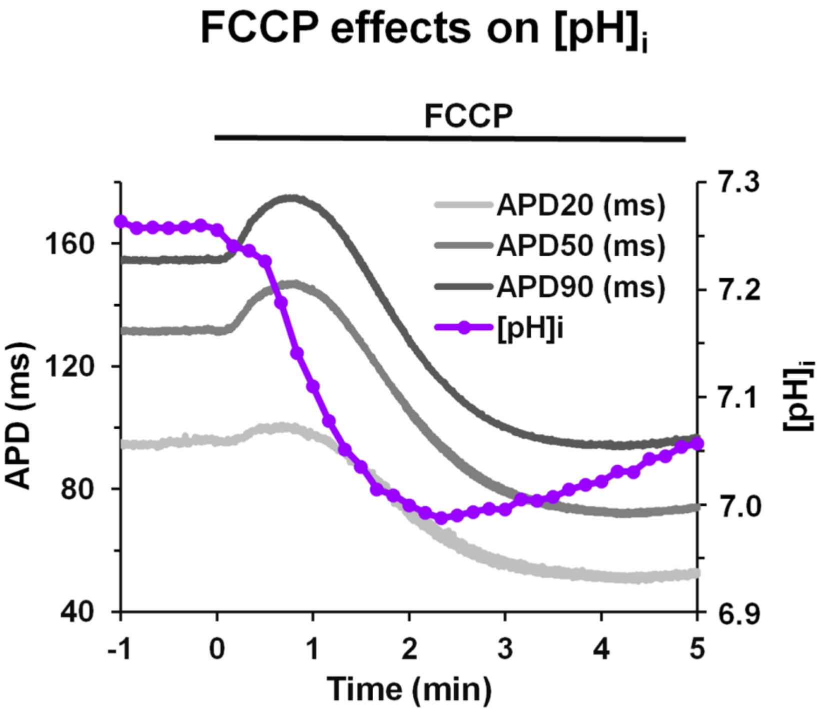

Figure S4

FCCP (1 µmol/L) induces intracellular acidification in Langendorff-perfused rabbit heart. Simultaneous recording of time-dependent fluorescence changes showing alterations of intracellular pH (pH_i, *violet*) and changes in microelectrode-recorded AP durations: APD20 (*light grey*), APD50 (*grey*) and APD90 (*dark grey*). Note that FCCP perfusion starts at time zero. Calibration of pH was conducted on a homogenized heart (in physiological solution). The SNARF-1 was added to the homogenate and the intensity of fluorescence was measured by changing pH. At the same time pH value was measured using a pH-meter.
