# Supplementary material for: Mechanism of Action Potential Prolongation During Metabolic Inhibition in the Whole Rabbit Heart
Source: Front Physiol. 2018 Aug 9;9:1077. doi: 10.3389/fphys.2018.01077 (PMC6095129; doi:10.3389/fphys.2018.01077)
Supplement: Supplementary file 6 [file Image_5.PDF]

Effect of FCCP on APD under blockade of I_{Kr} and I_{Ks}

To evaluate the possible impact of I_{Kr} and I_{Ks} on AP prolongation induced by metabolic inhibition, the hearts were pretreated with solution containing both blockers, i.e. E-4031 and HMR-1556, respectively, before FCCP application. Under blockade of I_{Kr} and I_{Ks}, APD20 prolonged by 34.55 ± 3.31%, APD50 – by 44.42 ± 1.55%, and APD90 – by 47.4 ± 1.0% (n = 4; $p < 0.05$ vs. control). The extra large prolongation of APs obliged us to increase pacing interval to 500 ms, therefore quantitatively the data became not comparable with the data obtained at 300 ms pacing interval. The typical example of time-dependent changes of microelectrode-recorded AP parameters and superimposed APs under effect of FCCP in the presence of E-4031 and HMR-1556 are shown in Figure S5. Note that the block of I_{Kr} and I_{Ks} did not eliminate FCCP-induced AP prolongation nor depolarization. Primarily, FCCP in the presence of both E-4031 and HMR-1556 did not influence APD20 but increased APD50 and APD90 by 11.18 ± 0.99 % and 12.87 ± 1.44%, respectively (n = 4; $p < 0.05$). Further, after AP prolongation the decrease of APD was observed in these conditions.

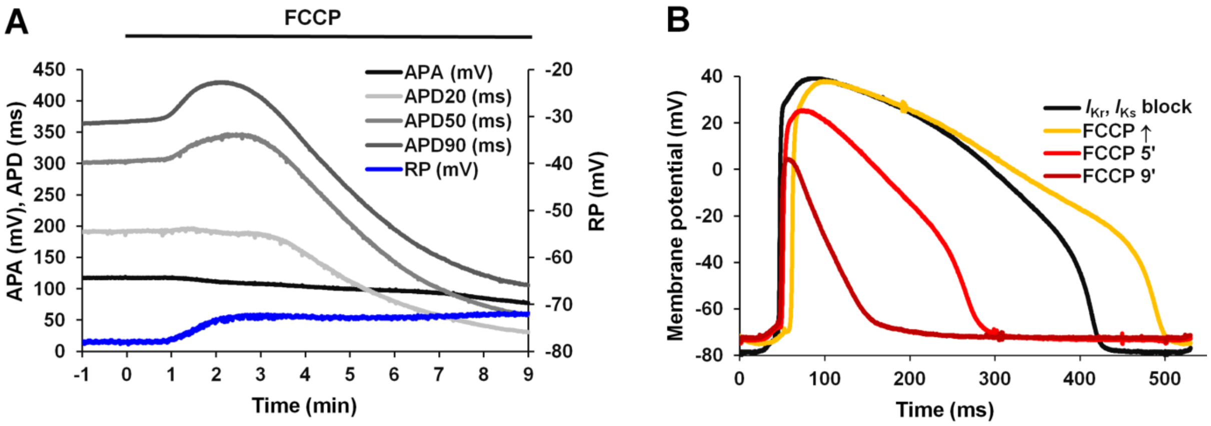

Figure S5

Effect of FCCP (1 µmol/L) on APD after pretreatment with I_{Kr} blocker E-4031 (10 µmol/L) and I_{Ks} blocker HMR-1556 (5 µmol/L) in Langendorff-perfused rabbit heart. **(A)** Typical example of time dependent changes in microelectrode-recorded APs: APA (*black*); APD20 (*light grey*), APD50 (*grey*), APD90 (*dark grey*), and RP (*blue*). FCCP perfusion started at time zero. **(B)** Superimposition of APs under blockade of I_{Kr} and I_{Ks} (*black*) and at FCCP↑ (*yellow*), at five minutes (*red*), and at nine minutes (*dark red*) of FCCP treatment.
